# Diffuse myocardial fibrosis is uncommon in people with perinatally acquired human immunodeficiency virus infection

**DOI:** 10.1186/s12981-024-00598-4

**Published:** 2024-03-04

**Authors:** Jason L. Williams, Frances Hung, Elizabeth Jenista, Piers Barker, Hrishikesh Chakraborty, Raymond Kim, Andrew W. McCrary, Svati H. Shah, Nathan Thielman, Gerald S. Bloomfield

**Affiliations:** 1 Division of Pediatric Cardiology, Duke University Medical Center, Durham, NC USA; 2 Department of Biostatistics and Bioinformatics, Duke University School of Medicine, Durham, NC USA; 3 Duke Cardiovascular Magnetic Resonance Center, Duke University Medical Center, Durham, NC USA; 4 Division of Cardiology, Duke University Medical Center, Durham, NC USA; 5 Duke Clinic Research Institute, Durham, NC USA; 6 Department of Radiology, Duke University Medical Center, Durham, NC USA; 7 Duke Clinic Research Institute and Duke University School of Medicine, Durham, NC USA; 8 Duke Clinic Research Institute and Duke University School of Medicine, Durham, NC USA; 9 Department of Medicine, Duke University School of Medicine, Durham, NC USA; 10 Division of Infectious Diseases and International Health, Department of Medicine, University Medical Center, Durham, NC USA; 11 Department of Medicine, Duke University Medical Center, DUMC Box 3850, 27705 Durham, NC USA; 12 Duke Clinical Research Institute, and Duke Global Health Institute, Duke University, Durham, NC USA

**Keywords:** Perinatal infection, HIV, Myocardial fibrosis

## Abstract

**Background:**

Cardiovascular disease (CVD) remains a leading cause of death in people living with HIV. Myocardial fibrosis is well-described in HIV infection acquired in adulthood. We evaluate the burden of fibrosis by cardiac magnetic resonance in people with perinatal HIV infection.

**Methods:**

Individuals with perinatally acquired HIV (pnHIV) diagnosed before 10 years-old and on antiretroviral treatment for ≥ 6 months were matched with uninfected controls. Patients with significant cardiometabolic co-morbidities and pregnancy were excluded. Diffuse fibrosis was assessed by cardiac magnetic resonance (CMR) with native T1 mapping for calculation of extracellular volume fraction (ECV). Viability was assessed with late gadolinium enhancement. The normality of fibrosis was assessed using the Komogrov-Smirnov test. Fibrosis between the groups was analyzed using a Mann-Whitney U test, as the data was not normally distributed. Statistical significance was defined as a p-valve < 0.05.

**Results:**

Fourteen adults with pnHIV group and 26 controls (71% female and 86% Black race) were assessed. The average (± standard deviation) age in the study group was 29 (± 4.3) years-old. All pnHIV had been on ART for decades. Demographic data, CMR functional/volumetric data, and pre-contrast T1 mapping values were similar between groups. Diastolic function was normal in 50% of pnHIV patients and indeterminate in most of the remainder (42%). There was no statistically significant difference in ECV between groups; *p* = 0.24.

**Conclusion:**

Perinatally-acquired HIV was not associated with diffuse myocardial fibrosis. Larger prospective studies with serial examinations are needed to determine whether pnHIV patients develop abnormal structure or function more often than unaffected controls.

## Introduction

Despite increased life expectancy with widespread use of antiretroviral therapy (ART), cardiovascular disease (CVD) remains a leading cause of mortality in people living with human immunodeficiency virus (PLWH) [1, 2]. Current data suggests that the burden of CVD is approximately 2-fold higher in PWH compared to the uninfected, and still remains a large, recognized risk in PLWH even when factoring in the pre-ART era into consideration [2]. Higher incidence is also seen in lower income regions, such as areas of sub-Saharan Africa (SSA) with the highest HIV burden, where CVD accounts for > 10% of all morbidity and mortality [3]. The pathophysiology for developing CVD is incompletely understood; however, etiologies include modifiable and unmodifiable risk factors, such as chronic disease inflammation, ART-associated dyslipidemia, early atherosclerosis, tobacco use, and inequitable care [2–4]. PLWH also have a greater degree of myocardial steatosis and fibrosis, and cardiovascular imaging has been increasingly used to define characteristics of subclinical CVD [5, 6].

Despite recent advances in knowledge about risk factors and prognostication of CVD in PLWH, there are sparse data from individuals with perinatally acquired HIV (pnHIV). Child death from HIV-associated cardiomyopathy has decreased; however, recent studies have shown both transient and chronic functional and structural changes by echocardiography [7, 8]. Cardiovascular magnetic resonance image (CMR) has emerged as a premier modality for assessment of CVD in PLWH, as it can detect abnormal structural, functional, and tissue characterization parameters better than other imaging modalities [4, 5, 9]. CMR has demonstrated fibrosis in assessment of late gadolinium enhancement (LGE) and mapping techniques in PLWH [4]. An increase in extracellular volume (ECV), a more sensitive marker for diffuse fibrosis, has also been described in this cohort. We are not aware of CMR studies that have focused specifically on adults with pnHIV. With this gap in mind, we sought to examine the myocardial tissue composition by CMR, specifically diffuse fibrosis, in patients with pnHIV.

## Methods

We enrolled persons with pnHIV, who were current or former patients at the Duke University Medical Center HIV clinic and included a historical control population for comparison. Inclusion criteria included age at diagnosis of less than 10 years-old with ability to provide consent, and administration of ART for at least ≥ 6 months duration, and ability to provide consent. Exclusion criteria included inability to provide consent, tolerate CMR examination, anaphylaxis to gadolinium contrast, recent medical illness requiring hospitalization in the prior 90 days, pregnancy (confirmed by urine pregnancy testing at the time of scan), breastfeeding, glomerular filtrate rate < 30 mL/min/m2, hemodialysis-dependent, and arrhythmia. The study was approved by the institutional review board at Duke University Medical Center, Pro00109701.

All newly enrolled participants underwent echocardiography or prior echocardiogram imaging was reviewed if obtained within 24 months. Study participants were age-matched with historic controls, who had no history of HIV infection or maternal exposure and who had undergone a CMR exam with sufficient LGE and ECV imaging. Enrolled and control group patients had no prior history of coronary artery disease, co-morbid inflammatory disease, chronic use of steroids or anti-inflammatory medication, active cancer, chemotherapy or radiation in the past year, myocardial infarction, moderate-severe valvular disease, congenital heart disease, heart failure, non-ischemic cardiomyopathy, atrial fibrillation, or intracardiac defibrillator/pacemaker placement. Common CMR indications for historical control patients included rule out cardiomyopathy and evaluation of chest pain.

CMR exams were performed on a 3T scanner (Siemens MAGNETOM Vida, Erlangen, Germany) in the Duke Cardiovascular Magnetic Resonance Center. Exams included functional analysis using ECG-gated steady-state free-precession (SSFP) cine imaging. Exams also included T2-weighted imaging for assessment of myocardial edema and native T1 mapping for diffuse fibrosis. Following intravenous administration of Gadolinium-based contrast agent (0.15 mmol/kg; Dotarem, Gurbet) late-gadolinium enhancement (LGE) images were acquired to assess for scar/fibrosis and post-contrast T1 mapping was performed to calculate extracellular volume (ECV).

Post-processing of CMR data was performed using Precession, HeartIT (Durham, NC) for function and volumetric analysis. Myocardial edema was assessed visually on T2-weighted images. T1 maps were quantitatively assessed using the inline color map provided by the scanner manufacturer (Siemens, Erlangen, Germany). Myocardial T1 relaxation time was manually measured from the maps by carefully contouring the endocardial and epicardial borders, excluding epicardial fat and blood pool [10]. LGE was scored for presence, location, and extent of hyperenhancement using the 17-segmental model and a 5-point sale as previously described [11].

The normality of fibrosis was checked using the Kolmogorov-Smirnov test. Fibrosis between the groups was analyzed using a Mann-Whitney U test as the data was not normally distributed. Statistical significance was defined as a p-valve < 0.05.

## Results

Fourteen patients were included in the pnHIV group (Table 1). Twenty-six patients without HIV infection were included as historic controls. Average (± standard deviation) age in the PWH group was 29 ± 4.3 years-old and the cohort was 71% female. Most patients were Black race (86%), and not of Hispanic or Latino ethnicity (100%). The majority of patients did not have hypertension, hyperlipidemia, diabetes or a smoking history in both groups. Comparison of demographic data revealed similar average values in age, body mass index, body surface area, systolic blood pressure, low-density lipoprotein, total cholesterol, hematocrit, creatinine and estimated glomerular filtration rate between groups. Those in the pnHIV group had slightly higher average diastolic blood pressure, high-density lipoprotein, and triglycerides. Most individuals in the pnHIV group (92%) reported no limitation of physical activity (i.e., New York Heart Association class 1). Excessive alcohol use or illicit drug use was uncommon.

**Table 1:** Demographic and clinical characterstics of the pnHIV and control groups

	pnHIV (*N* = 14)	Control, historic from cardiac MRI lab (*N* = 26)
Demographic and Clinical Data*		
Age (years)	29.1 [27.7, 31.6]	28.0 [25.0, 31.0]
Female Sex	10 (71.4%)	17 (65.4%)
Race		
Black/African-American	12 (85.7%)	22 (84.6%)
White	2 (14.3%)	3 (11.5%)
Unknown	0 (0%)	1 (3.8%)
BMI (kg/m2)	30.5 [24.5, 35.7]	30.4 [23.7, 34.5]
BSA (m2)	1.8 [1.7, 2.0]	1.9 [1.7, 2.1]
Hypertension	1 (7.1%)	3 (11.5%)
Missing	0	0
Dyslipidemia	0 (0%)	1 (3.8%)
Missing	0	2
Diabetes Mellitus	1 (7.1%)	0 (0%)
Missing	0	1
Myocardial Infarction	0 (0%)	0 (0%)
Missing	0	1
NYHA Class		
NYHA Class I	13 (92.9%)	--
NYHA Class II	1 (7.1%)	--
**Laboratory Data**		
eGFR (mL/min)	114.1 [92.3, 124.7]	93.3 [87.4, 119.3]
Missing	1	3
CD4 count (cells/uL)	632.5 [562.5, 1171.2]	--
Viral load (copies/mL)	0.0 [0.0, 23.0]	--
Missing	3	--
LV Mass Index (g/m2)	63.3 [54.8, 79.5]	--
Missing	0	--
Relative Wall Thickness (cm)	0.4 [0.3, 0.4]	--
LVIDd (cm)	4.5 [4.3, 4.7]	--
Calculated EF (%)	58.0 [55.0, 60.0]	--
Missing (n)	1	--
E/A Ratio	1.5 [1.4, 1.6]	--
E/E’ Ratio	6.6 [6.0, 8.0]	--
Global Longitudinal Strain (%)	-18.1 [-19.2, -17.2]	--
**CMR Data**		
T1 Pre-Contrast (milliseconds)	1230.1 [1220.1, 1242.9]	1235.4 [1210.4, 1256.0]
Missing	1	0
Extracellular Volume (%)	25.5 [24.1, 27.5]	27.5 [23.7, 28.8]
Missing	1	0
LV End-Diastolic Volume (mL)	139.7 [128.0, 147.0]	127.8 [114.1, 156.0]
Missing	1	0
LV End-Systolic Volume (mL)	58.8 [46.8, 60.8]	49.7 [43.1, 66.2]
Missing	1	0
LV Ejection Fraction (%)	58.4 [58.0, 63.8]	61.3 [58.4, 63.0]
Missing	1	0
LV Cardiac Output (Liters/minute)	5.6 [5.2, 6.0]	5.8 [5.2, 6.3]
Missing	1	0
LV Mass (grams)	96.5 [81.9, 104.7]	102.0 [82.5, 132.7]
Missing	1	0

*All data Median [Q1, Q3] or n(%)

**Fig. 1:**
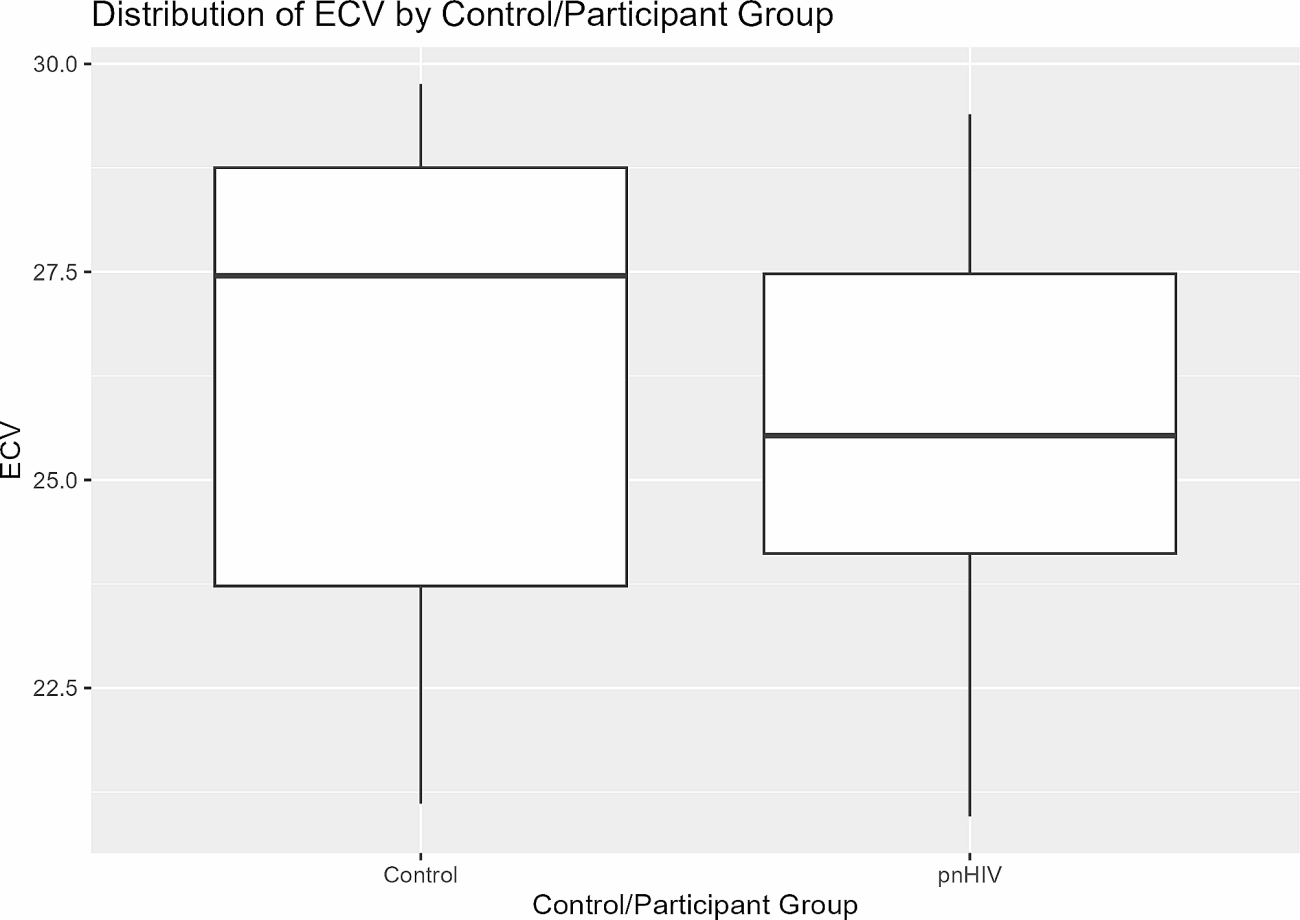
Extracellular volume in pnHIV and control groups

Echocardiographic data demonstrated normal structure and systolic function by both conventional functional assessment and subclinical myocardial strain analysis. Diastolic function was normal in 50% of the pnHIV group and indeterminate in 42%. Functional and volumetric data were similar between groups. Pre-contrast T1 mapping values were similar between groups. There was no statistically significant difference in ECV between groups; *p* = 0.24 (Fig. 1).

## Discussion

This is the first study to our knowledge to examine diffuse fibrosis in PLWH on ART, who acquired HIV perinatally. This group is of particular importance to study given three decades of HIV exposure, yet with few traditional CVD risk factors. Using historic controls to compare the prevalence of abnormal myocardial fibrosis, we found no statistically significant difference in ECV fraction between pnHIV group versus those unaffected by HIV.

As a part of CMR analysis in these patients, tissue characterization is performed through myocardial T1 and T2 mapping techniques, ECV calculation, and assessment of LGE by direct visualization. LGE is the gold standard for assessment of non-viable myocardium; however, LGE may not detect diffuse fibrosis due to lack of normal myocardium in extensive disease and limitations of spatial resolution [12]. ECV fraction is a marker of diffuse fibrosis, derived from native T1 values, shortened post-contrast T1 values (following administration of gadolinium-based contrast agents), and hematocrit. ECV provides an estimate of interstitial and extracellular matrix, which increases with ventricular remodeling and collagen deposition in disease states, such as myocarditis. CMR-based fibrosis in chronic HIV infection is well-established in patients that acquired HIV later in life [4].

Fibrosis in PLWH has important prognostic implications, including development of heart failure and death [13, 14]. Outcome studies in PLWH demonstrated higher prevalence of diffuse myocardial fibrosis by CMR in patients who had cardiac events compared to those that did not during short interval follow-up [14]. There was also statistically significant difference in myocardial edema and native T2 values in patients who had events compared to those who did not [14]. In our study, we found no differences in T1 mapping and ECV fraction between the patients with perinatal HIV from controls. Reasons are likely multifactorial and raises the question whether there are intrinsic differences between treated perinatal HIV infection and infection acquired later in life, as it relates to immune regulation, viral replication, antiretroviral exposures, and other mechanisms [15]. In a cross-sectional CMR-based study in South Africa, prolonged exposure to ART in perinatal infection is thought to be cardioprotective, causing less severe left ventricular remodeling, as evidenced by lower mass-to-volume ratio when compared to uninfected controls. No difference in tissue characterization was seen between groups [16]. Another possibility in our cohort is the low overall burden of fibrosis, which could yield non-significant difference in ECV. In a small prospective cohort study, LGE was seen in a majority of patients with HIV compared to controls; however, the overall volume of LGE was low at 3.4%. The authors suggested that the low extent of fibrosis, may also explain why there was nonsignificant difference in ECV fraction [17].

Our patient population with pnHIV is younger by decades than most published studies of PLWH who have undergone CMR to evaluate for fibrosis. Younger patients may lack traditional modifiable risk factors for early CVD, specifically dyslipidemia. In our study population, the prevalence of chronic metabolic conditions known to place patients at increased risk of CVD, such as obesity, diabetes mellitus, and hypertension, was low [17]. In addition, the majority of these patients had never smoked nor had coronary artery disease or myocardial infarction. When CVD risk factors are absent, adults with PLWH are less likely to have diastolic dysfunction by echocardiogram suggesting an important role of traditional CVD risk factors that may work in tandem with HIV to impair cardiac function [18]. While CVD in PLWH is likely related to viral infection, ART administration, and other co-morbidities, younger patients who have not acquired the compounded modifiable risks may lack CMR-based fibrosis.

Early and sustained exposure to ART is also likely to play a role in mitigating cardiac fibrosis. With pnHIV initiation of ART occurs sooner after infection than if infection acquired later life [19, 20]. Longer duration of ART results in less end-organ damage as the result of sustained undetectable viral load [21]. In a prospective study following serial CMR data in treatment naive patients with repeat exam after 9 months on ART, myocardial edema improved as evidenced by a decrease in native T1, T2 values, and ECV fraction [22]. Improvement in tissue composition was associated with a decrease in viral load and CRP, with an increase in CD4 cell count, suggesting that myocardial inflammation and edema are responsive to ART [22]. Improvement in inflammation supports that hypothesis that early initiation of ART in pnHIV improves cardiac tissue characteristics in these patients compared to infection acquired later.

There are several limitations to our study. This was a pilot study in an under-studied population that was limited in sample size. Our study may not have been sufficiently powered to detect difference in ECV, as it was difficult to recruit patients to undergo CMR examination during the COVID-19 epidemic. The patients recruited in our study are also likely to be the most compliant patients. As such, the findings may not be as generalizable to a broader context of PLWH. Our study was also a cross-sectional design, and did not prospectively follow patients longitudinally with serial CMRs to detect subtle changes in pre-contrast T1, LGE or ECV with time.

## Conclusion

No differences were detected in CMR-based markers of cardiac fibrosis among young adults with pnHIV and controls. Future direction should include serial assessment of tissue characterization over time, and compare pnHIV patients to both unaffected controls and patients who acquired infection later in life.

## Data Availability

The datasets used and/or analyzed during the current study are available from the corresponding author on reasonable request.
